# Predictors of pain in general ageing populations: results from a multi-country analysis based on ATHLOS harmonized database

**DOI:** 10.1186/s10194-020-01116-3

**Published:** 2020-05-06

**Authors:** Alberto Raggi, Matilde Leonardi, Blanca Mellor-Marsá, Maria V. Moneta, Albert Sanchez-Niubo, Stefanos Tyrovolas, Iago Giné-Vázquez, Josep M. Haro, Somnath Chatterji, Martin Bobak, Jose L. Ayuso-Mateos, Holger Arndt, Muhammad Z. Hossin, Jerome Bickenbach, Seppo Koskinen, Beata Tobiasz-Adamczyk, Demosthenes Panagiotakos, Barbara Corso

**Affiliations:** 1 Neurology, Public Health and Disability Unit, Fondazione IRCCS Istituto Neurologico Carlo Besta, Via Celoria 11, 20133 Milan, Italy; 2 Parc Sanitari Sant Joan de Déu, Fundacion Sant Joan de Deu, Barcelona, Spain; 3 Instituto de Salud Carlos III, Centro de Investigación Biomédica en Red de Salud Mental, CIBERSAM, Madrid, Spain; 4 Department of Nutrition and Dietetics, School of Health Science and Education, Harokopio University, Athens, Greece; 5 Information, Evidence and Research, World Health Organization, Geneva, Switzerland; 6 Research Department of Epidemiology and Public Health, University College London, London, UK; 7 Department of Psychiatry, Universidad Autónoma de Madrid, Madrid, Spain; 8 Hospital Universitario de La Princesa, Instituto de Investigación Sanitaria Princesa, Madrid, Spain; 9 SPRING TECHNO GMBH & Co. KG, Bremen, Germany; 10 Department of Global Public Health, Karolinska Institute, Stockholm, Sweden; 11 Department of Health Sciences and Health Policy, University of Lucerne, Lucerne, Switzerland; 12 Swiss Paraplegic Research, Nottwil, Switzerland; 13 Finnish Institute for Health and Welfare (THL), Helsinki, Finland; 14 Department of Epidemiology and Population Studies, Faculty of Health Sciences, Jagiellonian University Medical College, Krakow, Poland; 15 National Research Council, Neuroscience Institute, Padova, Italy

**Keywords:** Pain, Risk factors, Headache disorders, Musculoskeletal disorders, Sleep, Obesity, Exercise, Bereavement, Fatigue, Walking

## Abstract

**Background:**

Pain is a common symptom, often associated with neurological and musculoskeletal conditions, and experienced especially by females and by older people, and with increasing trends in general populations. Different risk factors for pain have been identified, but generally from studies with limited samples and a limited number of candidate predictors. The aim of this study is to evaluate the predictors of pain from a large set of variables and respondents.

**Methods:**

We used part of the harmonized dataset of ATHLOS project, selecting studies and waves with a longitudinal course, and in which pain was absent at baseline and with no missing at follow-up. Predictors were selected based on missing distribution and univariable association with pain, and were selected from the following domains: Socio-demographic and economic characteristics, Lifestyle and health behaviours, Health status and functional limitations, Diseases, Physical measures, Cognition, personality and other psychological measures, and Social environment. Hierarchical logistic regression models were then applied to identify significant predictors.

**Results:**

A total of 13,545 subjects were included of whom 5348 (39.5%) developed pain between baseline and the average 5.2 years’ follow-up. Baseline risk factors for pain were female gender (OR 1.34), engaging in vigorous exercise (OR 2.51), being obese (OR 1.36) and suffering from the loss of a close person (OR 1.88) whereas follow-up risk factors were low energy levels/fatigue (1.93), difficulties with walking (1.69), self-rated health referred as poor (OR 2.20) or average to moderate (OR 1.57) and presence of sleep problems (1.80).

**Conclusions:**

Our results showed that 39.5% of respondents developed pain over a five-year follow-up period, that there are proximal and distal risk factors for pain, and that part of them are directly modifiable. Actions aimed at improving sleep, reducing weight among obese people and treating fatigue would positively impact on pain onset, and avoiding vigorous exercise should be advised to people aged 60 or over, in particular if female or obese.

## Introduction

Pain is one of the most common symptoms in general populations and is the core symptom of many common, and often comorbid, clinical conditions. In fact, the prevalence of pain-associated conditions is between 10% and 60% approximately, with the highest rates being observed for tension-type headache [1–11]. Data referred to general populations are on the contrary almost lacking. A recent study from the U.S. showed that the overall rates of non-cancer pain prevalence increased from 32.9% to 41.0% over an 18-years period (1997–98 to 2013–14) [12]. Such an information was confirmed in a recent study based on the harmonized dataset generated by ATHLOS project (Ageing Trajectories of Health – Longitudinal Opportunities and Synergies) [13]. Such a recent paper showed that pain rates largely vary by sex, participants’ age, period of inclusion in the studies and birth cohort [14]. In particular, pain rates were shown to vary between 20 and 30% in males enrolled in the early 1990s, and 60–70% among old-age women enrolled between 2011 and 2015. In addition to this, the 10-years forecast suggested an increase in the trends of pain which will peak up to 20% among females and among older subjects [14].

Given the observed and predicted increase of pain rates, determinants need to be addressed in order to limit to the widest extent the possible negative consequences of such an expansion, for example the risk of future opioids and other drugs’ overuse, increase in disability, reduction in quality of life and employment rates, and productivity loss [15–25]. A large amount of studies identified potential predictors of pain, both in clinical trials and in population or cohort studies. Among population and cohort studies, evidence existed on the impact of sociodemographic characteristics, with females [26–33] and older people [6, 27, 28, 31, 32, 34] reporting higher pain rates or severity. Also, people with lower education level and those unemployed had higher pain rates [35, 36], but contrasting results were found with reference to socioeconomic position: in fact, Chen and colleagues found that people with a lower socioeconomic class had a more severe trajectory with regard to low back pain severity and persistence [37], whereas Elsharydah and colleagues found that higher median household income was associated with higher rate of complex regional pain syndrome type 1 [30]. Evidence was also found on the impact of some mental health problems and symptoms on pain rates and severity, including sleep problems [6, 27, 34, 38, 39], depressed mood or anxiety [26, 28–30, 34, 35, 39, 40], cognitive complaints [34] and fatigue or lack of energy [38]. Other health status variables that were found to be associated with higher pain rates and severity included the presence of comorbidities and multimorbidity status [6, 26, 30, 35, 39, 41] and, among single heath conditions, diabetes and stroke [30, 42, 43]: of course, presence of conditions whose cardinal symptom is pain, such as headaches, musculoskeletal conditions or angina, is clearly acknowledged to impact on pain. Finally, a set of risk and protective lifestyle factors were also identified. Physical activity was found to be protective against back pain onset among older adult women [29], smoking was found to be a risk factor for the development of chronic musculoskeletal pain [44], and high body mass index (BMI) or obesity status were risk factors for higher pain rates, development of musculoskeletal conditions and pain worsening [6, 27, 29, 30, 33, 35, 45, 46].

The information herein available is however limited to specific cohorts, such as military or farmers, or cohort of patients, with population studies presenting a limited amount of pain predictors, a relatively limited amount of participants, with different ages and a limited geographical distribution. This provides a partial appreciation of global pain predictors, which can be on the contrary achieved through an analysis of a large dataset such as that of ATHLOS project, where data from 17 different population survey conducted in the five continents were harmonized [13]. This paper aims to provide the most comprehensive identification of pain predictors in ageing populations.

## Methods

### Study population

The last available wave of each study (i.e. harmonized in the ATHLOS project) was defined as *follow-up* wave, while the *baseline* wave was selected trying to keep, when possible, one wave in between the two. The baseline wave did not necessarily coincide with study’s baseline wave.

The studies and waves selected for the present analyses were: the China Health and Retirement Longitudinal Study (CHARLS: W1-W2), the Collaborative Research on Ageing in Europe (COURAGE in Europe: W1-W2), the Health and Retirement Study (HRS: W9-W11), the Health 2000/2011 study (W1-W2), the Mexican Health and Aging Study (MHAS: W2-W3), and the Survey of Health, Ageing and Retirement in Europe (SHARE: W4-W5). On average, the follow-up waves were carried out 5.2 (SD 3.2) years after the baseline waves (see Table S1 in supplementary materials for pain variable distribution and follow-up duration in the different studies).

### Variables of interest

The outcome variable was the absence/presence of the harmonized pain variable (defined as “self-reported pain experienced at the time of the interview”) at follow-up. Absence of pain was defined as absence of pain at both baseline and follow-up wave, while presence of pain was identified as absence of pain at baseline but presence of pain at follow-up. In total, 91,278 subjects did not report pain at baseline and, of them, 50,849 had complete data on pain at follow-up: of them 36,023 still did not report pain, whereas 14,826 (29.2%) reported pain.

Candidate predictors of pain were selected from the different domains defined in the ATHLOS harmonization. Domain “Lifestyle and health behaviours”: current smoking status, current alcohol use and engagement in vigorous exercise; “Health status and functional limitations”: level of energy, sleep quality, mobility walk, self-reported health, presence of recent falls and evaluative wellbeing; “Diseases”: diabetes, respiratory disease, hypertension, joint disorders, angina, stroke, cancer and multimorbidity; “Physical measures”: obesity; “Cognition, personality and other psychological measures”: depression; “Social environment”: bereavement, i.e. the experience of a loss of any close person. Moreover, in the domain “Socio-demographic and economic characteristics”, gender, marital status, education, retirement status and wealth quintile of participants were retrieved. All these variables were selected from both baseline and follow-up waves (see Table S2 in supplementary materials for the definition of harmonized variables).

### Data analysis

Initially, as a screening criterion, we excluded variables with a relevant amount of missing that would have dramatically reduced the sample size exploitable for the present analysis. Second, predictors of pain were assessed using univariable logistic regression models and those with a *p*-value< 0.10 were retained in the subsequent analyses (see Table S2 in supplementary materials).

Next, two stepwise forward logistic regressions were performed (the *p*-value for variable inclusion and removal were set to *p* < 0.05 and *p* < 0.15, respectively). The first stepwise was performed with baseline variables only as predictors of pain development at follow-up, and later a multivariable logistic regression was implemented retaining only statistically significant predictors (*p* < 0.05). Then, in the second stepwise, the significant predictors identified at baseline were kept in the model and follow-up variables were included for selection. Lastly, a final model was pursued with only significant predictors at both baseline and follow-up. All models were weighted and adjusted for a time-lag variable (defined as the difference between the year of interview at follow-up and baseline). Multicollinearity was checked using tolerance and Variance Inflation Factor (VIF): variables with tolerance < 0.4 (VIF > 2.5) were discarded from the analysis. Engagement in vigorous exercise at follow-up was discarded form the analyses due to collinearity. Models’ goodness-of-fit were assessed by the Hosmer and Lemeshow’s test and by visual inspection of the plot of estimated values against residuals, whilst specification error was evaluated by running a new regression with the observed values against predicted and predicted-squared values as independent variables. The Area Under the Receiver Operating Curve (AUC) and its 95% confidence interval (95% CI) for the predicted versus the actual data was also calculated. Categorical variables were reported as proportions, and continuous variables were reported as means ± standard deviations (SD) and medians with interquartile ranges (IQRs). Odds ratios (ORs) were presented with their 95% CI and Z with its associated *p*-value. All statistical analyses were performed using STATA SE, version 15.0 (Stata Statistical Software: Release 12. College Station, TX: StataCorp LP).

## Results

Almost all variables, at both baseline and follow-up, were significant predictors of pain in univariable regression models (see Table S2 in supplementary materials): the exceptions to this were being single (vs. married) at baseline and being retired (vs. employed or student) at follow-up.

Stepwise regression with baseline variables enabled retaining eight predictors (gender, sleep problems, engagement in vigorous exercise, bereavement, obesity, self-rated health, multimorbidity and smoking), and follow-up variables enabled retaining seven predictors (energy level, difficulties with walking, respiratory diseases, joint disorders, stroke and again self-rated health and sleep problems). The full results of multivariable regression models are in supplementary materials (see Table S3). The final sample size was composed of 13,545 subjects, 8197 without pain and 5348 that developed pain between baseline and follow-up, corresponding to 39.5% of the sample.

Table 1 provides full features of the final sample. Females represented 53.3% of the entire sample, and average age moved from a median of 63 to a median of 68 years, and more than one-third of the sample was retired. Most descriptive variables were almost similar at the two time points, with some exceptions: for example, difficulty walking increased from 9.7% to 18.7%, and poor self-reported health from 5.1% to 8.9%. Among health conditions, hypertension and joint disorders were the two most common at baseline (37.4% and 26.6%, respectively) and underwent a significant increase (51.6% and 38.6% at follow-up, respectively), and multimorbidity was experienced by 27.3% of participants at baseline and 40.1% at follow-up. The same data are reported study by study in supplementary materials (see Tables S4, S5, S6, S7, S8, S9).

**Table 1:** Final regression model sample description

	Baseline			Follow-up		
	No pain at follow-up (*N* = 8197)	Pain at follow-up (*N* = 5348)	Total (*N* = 13,545)	No pain at follow-up (*N* = 8197)	Pain at follow-up (*N* = 5348)	Total (*N* = 13,545)
Age, mean ± sd [median: interquartile range]	59.8 ± 14.4 [64: 52–71]	60.62 ± 13.0 [62: 53–70]	60.1 ± 13.9 [63: 53–70]	65.5 ± 13.9 [70: 59–75]	64.9 ± 12.6 [66: 57–74]	65.3 ± 13.5 [68: 57–75]
Sex			(*N* = 13,545)			(*N* = 13,545)
Male	49.8%	41.9%	46.7%	49.8%	41.9%	46.7%
Female	50.2%	58.1%	53.3%	50.2%	58.1%	53.3%
Marital status			(*N* = 13,529)			(*N* = 13,526)
Married/cohabiting	71.1%	71.4%	71.2%	67.2%	67.2%	67.2%
Single	8.3%	8.0%	8.2%	7.5%	7.8%	7.6%
Divorced/Separated	7.1%	7.1%	7.1%	7.7%	7.3%	7.6%
Widow	13.5%	13.5%	13.5%	17.6%	17.7%	17.6%
Education			(*N* = 11,384)			(*N* = 9931)
Primary or less	28.6%	38.3%	32.5%	20.3%	31.6%	24.9%
Secondary or above	71.4%	61.7%	67.5%	79.7%	68.4%	75.1%
Retired			(*N* = 12,984)			(*N* = 12,974)
	31.2%	34.3%	32.5%	41.9%	40.5%	41.3%
Household wealth quintile			(*N* = 13,140)			(*N* = 10,175)
1 - Lower	15.7%	20.0%	17.4	19.5%	23.3%	21.0%
2	17.6%	19.0%	18.2	18.4%	21.7%	19.7%
3	19.7%	17.9%	19.0	20.1%	19.7%	19.9%
4	23.5%	22.3%	23.0	21.1%	17.5%	19.7%
5 - Higher	23.5%	20.8%	22.4	20.9%	17.8%	19.7%
Smoking status,			(*N* = 12,674)			(*N* = 10,629)
Never	47.4%	51.4%	48.9%	44.0%	44.2%	44.0%
Former smoker	31.7%	25.1%	29.3%	39.8%	30.1%	36.9%
Current	20.9%	23.5%	21.8%	16.2%	25.7%	19.1%
Current alcohol consumption			(*N* = 13,541)			(*N* = 13,008)
	57.2%	56.8%	57.1%	55.5%	56.0%	55.7%
Engaged in vigorous exercise			(*N* = 13,545)			(*N* = 11,901)
	25.6%	38.7%	30.7%	21.4%	33.7%	26.3%
Low level of energy/Fatigue			(*N* = 13,413)			(*N* = 13,545)
	25.7%	34.0%	28.9%	21.6%	43.8%	30.3%
Sleep problems			(*N* = 13,430)			(*N* = 13,545)
	24.8%	35.9%	29.1%	25.8%	45.1%	33.3%
Difficulty walking by yourself			(*N* = 12,972)			(*N* = 13,545)
	8.4%	11.6%	9.7%	14.1%	25.9%	18.7%
Self-reported health			(*N* = 12,818)			(*N* = 13,545)
Poor	3.5%	7.5%	5.1%	4.8%	15.1%	8.9%
Average/Fair/Moderate	19.6%	27.2%	22.6%	21.7%	34.6%	26.7%
Good	76.9%	65.3%	72.3%	73.5%	50.3%	64.4%
Recent falls			(*N* = 9656)			(*N* = 10,092)
	26.2%	64.6%	42.4%	30.7%	68.2%	46.3%
Evaluative wellbeing,			(*N* = 7178)			(*N* = 9467)
Low	5.7%	22.2%	14.4%	6.3%	25.0%	15.2%
Middle	20.8%	27.9%	24.5%	18.6%	28.2%	23.2%
High	73.5%	49.9%	61.1%	75.1%	46.8%	61.6%
Diabetes			(*N* = 11,928)			(*N* = 12,214)
	11.8%	15.7%	13.1%	19.0%	19.6%	19.2%
Respiratory diseases			(*N* = 13,531)			(*N* = 12,084)
	8.0%	9.2%	8.5%	10.7%	14.0%	12.0%
Hypertension			(*N* = 13,527)			(*N* = 12,609)
	37.5%	37.4%	37.4%	50.8%	52.8%	51.6%
Joint disorders			(*N* = 13,537)			(*N* = 12,688)
	26.6%	26.7%	26.6%	347%	44.4%	38.6%
Angina			(*N* = 3453)			(*N* = 3453)
	1.5%	2.2%	1.7%	2.8%	4.5%	3.4%
Stroke			(*N* = 13,535)			(*N* = 12,084)
	1.1%	1.9%	1.4%	1.7%	2.7%	2.1%
Cancer			(*N* = 11,922)			(*N* = 11,503)
	1.7%	3.2%	2.3%	2.3%	4.6%	3.2%
Multimorbidity			(*N* = 13,544)			(*N* = 13,096)
	27.6%	27.0%	27.3%	38.4%	42.6%	40.1%
Obesity			(*N* = 13,545)			(*N* = 11,245)
	19.0%	23.1%	20.6%	21.2%	24.5%	22.5%
Depression			(*N* = 13,302)			(*N* = 13,451)
	10.6%	22.3%	15.2%	10.6%	29.8%	18.1%
Experience of a loss of any close person (bereavement)			(*N* = 13,545)			(*N* = 13,536)
	28.6%	44.5%	34.8%	33.4%	58.3%	43.1%

Table 2 reports the results of the following models: Model 1, stepwise-selected baseline predictors; Model 2, significant baseline predictors only (*p* < 0.05); Model 3, significant baseline predictors and stepwise-selected follow-up predictors. All baseline variables, with the exclusion of smoking status and multimorbidity, were significant risk factors for pain at 5.2 years’ follow-up. Once variables selected at follow-up were included, baseline self-rated health was no more significant, whereas the corresponding follow-up variable was. Among newly included variables, low-level energy/fatigue, difficulties with walking, sleep problems and respiratory disease were significant risk factors for pain occurrence, whereas neither joint disorders nor stroke were significant predictors.

**Table 2:** Hierarchical pain predictive models: stepwise-selected baseline predictors, significant baseline predictors only (*p* < 0.05), significant baseline predictors and stepwise-selected follow-up predictors

	Model 1: stepwise-selected baseline predictors (*N* = 16,097)		Model 2: baseline with significant predictors only (*N* = 16,979)		Model 3: significant baseline predictors and stepwise-selected follow-up predictors (*N* = 11,852)	
	OR (95% CI)	Z (*p*-value)	OR (95% CI)	Z (*p*-value)	OR (95% CI)	Z (*p*-value)
Baseline Variables						
Female gender	1.39 (1.24–1.56)	5.51 (<.001)	1.42 (1.28–1.58)	6.49 (<.001)	1.36 (1.18–1.56)	4.34 (<.001)
Engage in vigorous exercise	1.50 (1.32–1.71)	6.10 (<.001)	1.73 (1.53–1.95)	8.93 (<.001)	3.10 (2.64–3.64)	13.69 (<.001)
Bereavement	1.31 (1.17–1.46)	4.59 (<.001)	1.50 (1.35–1.67)	7.44 (<.001)	1.90 (1.64–2.20)	8.52 (<.001)
Obesity	1.23 (1.09–1.38)	3.45 (.001)	1.22 (1.09–1.36)	3.37 (<.001)	1.17 (1.00–1.36)	1.97 (.049)
Self-rated health – average to moderate (vs. good)	1.65 (1.44–1.89)	7.23 (<.001)	1.75 (1.54–1.99)	8.64 (<.001)	1.07 (0.89–1.28)	0.68 (.499)
Self-rated health – poor (vs. good)	2.24 (1.78–2.81)	6.97 (<.001)	2.26 (1.82–2.81)	7.35 (<.001)	0.95 (0.61–1.48)	−0.23 (.819)
Sleep problems	1.35 (1.21–1.52)	5.13 (<.001)	1.41 (1.26–1.57)	5.98 (<.001)	1.25 (1.07–1.46)	2.78 (.005)
Multimorbidity	1.10 (0.98–1.24)	1.60 (.109)	–	–	–	–
Smoking – Former smoker (vs. never smoker)	0.87 (0.77–1.00)	−2.02 (.043)	–	–	–	–
Smoking – Current smoker (vs. never smoker)	1.15 (0.98–1.24)	1.91 (.056)	–	–	–	–
Follow-up Variables						
Low energy level/Fatigue					1.97 (1.67–2.32)	8.15 (<.001)
Self-rated health – average to moderate (vs. good)					2.32 (1.94–2.78	9.19 (<.001)
Self-rated health – poor (vs. good)					3.80 (2.57–5.60)	6.71 (<.001)
Difficulties with walking					1.52 (1.26–1.83)	4.35 (<.001)
Sleep problems					1.88 (1.61–2.18)	8.13 (<.001)
Respiratory disease					1.26 (1.03–1.56)	2.19 (.028)
Joint disorders					0.96 (0.83–1.11)	−0.57 (.571)
Stroke					0.90 (0.54–1.52)	−0.38 (.702)

Notes. Model 1: Hosmer-Lemeshow’s *p*-value = .085; AUC 0.64 (95% CI: 0.63–0.65). Model 2: Hosmer-Lemeshow’s *p*-value = .038; AUC 0.65 (95% CI: 0.64–0.66). Model 3: Hosmer-Lemeshow’s *p*-value = .010; AUC 0.79 (95% CI: 0.78–0.80)

Table 3 shows the results of the final model, where significant predictors only were retained. Being female, engaging in vigorous exercise, being obese and bereavement were all risk factors for the onset of pain over an average period of 5.2 years. In addition to this, low energy levels, difficulties with walking, non-good self-rated health and presence of sleep problems were significant risk factors for the presence of pain at the follow up.

**Table 3:** Final hierarchical pain predictive model

	Model 4: significant baseline and follow-up predictors (*N* = 13,545)	
	OR (95%CI)	Z (*p*-value)
Baseline Variables		
Female gender	1.34 (1.18–1.51)	4.61 (<.001)
Engage in vigorous exercise	2.51 (2.18–2.89)	12.71 (<.001)
Bereavement	1.88 (1.65–2.15)	9.39 (<.001)
Obesity	1.36 (1.18–1.57)	4.20 (<.001)
Follow-up Variables		
Low energy level/Fatigue	1.93 (1.68–2.20)	9.27 (<.001)
Self-rated health – average to moderate (vs. good)	1.57 (1.36–1.82)	6.04 (<.001)
Self-rated health – poor (vs. good)	2.20 (1.71–2.83)	6.13 (<.001)
Difficulties with walking	1.69 (1.43–1.99)	6.12 (<.001)
Sleep problems	1.80 (1.58–2.05)	8.97 (<.001)

Note: Hosmer-Lemeshow’s *p*-value = .001; AUC 0.77 (95% CI 0.76–0.78)

## Discussion

The results of this study, based on a large sample of general ageing population, showed that 39.5% of respondents developed pain over a five-year follow-up period and that there are proximal and distal risk factors for pain. Proximal risk factors include low energy level (or fatigue), walking difficulties, self-rated health perceived as not good and sleep problems, whereas distal risk factors – which predicted pain onset over a five-year timeframe – included female gender, engaging in vigorous exercise, being obese and suffering from the loss of a close person. It is interesting to notice that two risk factors, namely sleep problems and poorer self-rated health were retained among distal ones, but they were discarded from the final model once proximal risk factors were included.

What makes our results of such an importance is that most of the risk factors herein included are modifiable, either directly or indirectly. Factors that may be directly addressed include sleep problems, obesity, engagement in vigorous activities, low energy/fatigue and walking difficulties, whereas factors such as self-rated health can be targeted through general health improvement interventions, as well as through interventions aimed to address some of the aforementioned targets, such as sleep improvement and weight loss. In addition to this, we did not include age as a variable in our analysis, but decided to include the ageing process itself, by weighting and adjusting all our models for a time-lag variable, i.e. the years passing between the two time points in each study.

Sleep problems may impact on pain in different ways, and are part of common associated symptomatology in different pain-related conditions affecting adults and older adults, such as low back pain, headache disorders or osteoarthritis [47–49]. As reported in a recent review [50] several hypotheses have been made. Disrupted sleep continuity (i.e. wakefulness during the night) may contribute to increased pain perception (and higher opioids consumption) through disruption of opioid circuits involved in the descending pain modulatory systems, and involvement of inflammatory mechanisms typical of ageing-related conditions such as cardiovascular disorders, hypertension and diabetes, has also been addressed. In fact, sleep disturbance were associated with increases in markers of systemic inflammation, such as interleukin-6, which is known to sensitize nociceptors and increase pain sensitivity [51]. It has to be acknowledged that insomnia and reduced sleep hours are part of the ageing process itself, with older people having difficulty falling and staying asleep due to frequent arousals [52]. Treatments are both pharmacological and non-pharmacological [53–55]: among the first, benzodiazepines, sedating low-dose antidepressants, antipsychotics and anticonvulsants are included, whereas non-pharmacological treatments mostly include cognitive behavioural therapy. There is a growing interest on behavioural treatment for sleep disturbances in older adults and elderlies, mostly in reason of their reduced risks and hazards of sedative-hypnotics and opioids for middle-aged and older adults, as well as in consideration of the interaction between drugs prescribed for sleep problems and those for other medical conditions [51, 53].

Obesity is another known factor contributing to pain, through both inflammatory and mechanical processes [56, 57]. Obesity is in fact a pro-inflammatory condition and literature exists on the role of proinflammatory cytokines in producing a hyperalgesic state. Excess of adipose tissue may in fact lead to an increased inflammatory response with through different chemical mediators involved in inflammation. Parallel to this, obesity determines increased risk and severity of musculoskeletal conditions, such as back pain [58]. In fact, the overload over low back, hip and knee joints cause injury and degradation to the cartilage and bone matrix in these structures, leading to osteoarthritis. In addition to this, known comorbidities of obesity that might exacerbate pain have to be taken into account, in particular sleep disorders (e.g. obstructive sleep apnoea) and depression [57, 59–61]. Evidence exist that weight loss programs, which include diet therapy and physical activity or, in specific cases, bariatric surgery, have positive effect on pain reduction in patients with different types of chronic musculoskeletal pain [62], osteoarthritis [63], back and knee pain [64], and migraine [65], and it showed to reduce up to 50% the risk of developing osteoarthritis on a 10-year period [66].

Exercising is protective for health in general, as also stated in the recent consensus statement on physical activity and ageing [67] where, however, an explicit mention to the fact that benefits for health among older adults can be realised at lower volumes and intensity than with the usually recommended “150min of moderate to vigorous intensity physical activity per week” is made. Besides exposing people to a higher risk of sport-related injuries, moderate to vigorous exercise is predictive of the onset of back pain among ageing populations [58, 68], worsening of osteoarthritis-related pain severity [69]. Therefore, older adults and aging population should engage in mild to moderate, rather than vigorous, exercise.

Pain and fatigue have been found to be connected in several conditions, in particular musculoskeletal disorders such as rheumatoid arthritis and neuromuscular disorders such as fibromyalgia and chronic fatigue syndrome, and it is besides associated to poor sleep and symptoms of anxiety and depression [70–73]. However, the way in which fatigue exerts its effect on pain is unclear, but some hypotheses have been postulated, which include a common pathway mediated by inflammatory processes, such as increased levels of pro-inflammatory cytokines, and sensitisation of muscle nociceptors [72, 74]. Treatment strategy for fatigue include nutraceuticals, although with conflicting results [75–77], and exercise therapy [78].

Walking difficulties can be the outcome of several health problems – such as stroke, obesity or osteoarthritis – and thus require disease-specific treatments. For example, in post-stroke patients gait difficulties are very common and generally due to impairments in muscle strength, muscle tone, control over voluntary movement and balance, which result in reduced walking speed, temporal and spatial inter-limb asymmetries or impaired balance control [79]. Therefore, approaches to gait and walking rehabilitation in stroke survivors will need to address different impairments, making it really a patient-specific treatment. Among obese subjects, walking impairments are often due to knee osteoarthritis [80] and respiratory impairment or comorbidities [81, 82], and rehabilitation programmes for obesity reduction include moderate exercise aimed to improve walking, with positive effect on pain outcomes [62–64].

Conversely, our final model did not include some risk factors that have been found in previous literature, including sociodemographic data such as education and unemployment [35, 36], risk factors such as smoking [44], and health condition such as depression, diabetes, stroke, musculoskeletal diseases and multimorbidity status [6, 26, 28–30, 32, 34, 35, 39–41]. Some of them, namely smoking status vs. never smoking, multimorbidity, joint disorders or stroke, were included in the model, but not retained when other variables were included or when the final model with significant predictors only was run. The reason for this lack of predictive power is likely due to the large amount of variable we used, most of which were significantly associated in univariable analyses (see Table S2 in supplementary materials). A separate note has to be made for depression which was not selected from the stepwise logistic regression. Our data showed in fact that bereavement, i.e. the loss of a close person, was predictive of pain over a five-year period, whereas depression was not. Some studies showed that in some bereaved individuals the death of a loved one precipitates a combination of symptoms of both grief and depression, as well as of traumatic stress, although the categories are not overlapping [83–85], and depression was found in 18% to 55% of bereaved people [86–88]. Our hypothesis is that in a sample of ageing population like the one we analysed – where one-third of participants at baseline reported the experience of the loss of a close person, and 15% reported depression – the experience of bereavement confounded and overcame the predictive power of depression towards pain prediction. In addition to this, have no indication on the amount of time that passed between the loss experience and the interview, and on the presence of a problematic grief among bereaved people in our sample.

Some limitations have to be acknowledged. First, the variables used for our analysis are from a harmonized dataset, with a clear simplification from their original formulation. The main outcome definition is “self-reported pain experienced at the time of the interview”, with a dichotomous output, and, for most of the studies herein used, the original items addressed pain in terms of severity and impact of pain, which spans between mild and disabling. Another variable for which relevant differences exist is “energy level” which in some studies (e.g. COURAGE in Europe) was addressed in terms of the amount of energy the person had (with response options varying between “not at all” and “completely”) [89], whereas other studies (e.g. CHARLS) addressed it in terms of fatigue or “feeling that everything is an effort” (with response options based on frequency, from “rarely” to “most or all of the time”) [90]. Second, there is a considerable variation in the lag between waves across studies, which varied between 2 and 11 years: we included the lag variable so to interpret predictors controlling for the difference between respondents’ ageing across studies. Third, stepwise regression, which had the merit to enable reducing the amount of predictors to significant ones, present intrinsic problems, in particular biased high R^2^ values, small standard error and narrow 95% CI for odd ratios, small and difficult to correct *p*-values due to multiple comparisons and exacerbated collinearity problems [91]. Fourth, when we selected the variables to be included in the models, we had to face the problems of variable missing values, which prevented some of them from being included (e.g. the variables “History of angina” and “Myocardial infarction or heart attack” had 51% and 55% of missing, respectively, whereas “recent falls” which his intuitively of importance had 47% of missing). Possible solutions preserving to loose sample would have included treating them as a separate category by itself and data imputation. The problem with the first is that it would have led to biased estimation in regression coefficients. Imputation of missing values, e.g. through mean imputation or regression-based imputation, was not considered in reason of the very limited set of records, namely around 2000 out of the 50,849 not referring pain at baseline and with complete pain information at follow-up, with complete information for the remaining variables of interest: the reliability of a model with such a large amount of imputed values would have been at least debatable.

## Conclusions

In conclusion, we showed that over a five-year period 39.5% of respondents from this ageing population that did not experience pain at baseline, developed pain at follow-up, and that there are distal and proximal risk factors for new pain onset. The first group included female gender, engaging in vigorous activities, being obese and suffering from the loss of a close person, whereas the second included low energy level (or fatigue), walking difficulties, self-rated health perceived as not good and sleep problems. Part of these predictors are directly modifiable: therefore, it is expectable that actions aimed at improving sleep, reducing weight among obese people and treating fatigue would positively impact on pain onset. In addition, indication to avoid vigorous exercise should also be provided to people aged around 60 years or older, in particular if female or obese.

## Supplementary information

**Additional file 1: Table S1.** Description of exploited studies and waves. **Table S2.** Univariable logistic regression results. **Table S3.** Multivariable logistic regression results. **Table S4.** Descriptive statistics, China Health and Retirement Longitudinal Study (CHARLS). **Table S5.** Descriptive statistics Collaborative Research on Ageing in Europe Study (COURAGE in Europe). **Table S6.** Descriptive statistics Health and Retirement Study (HRS). **Table S7.** Descriptive statistics Health 2000/2011 study (Health 2000/2011). **Table S8.** Descriptive statistics Mexican Health and Aging Study (MHAS). **Table S9.** Descriptive statistics Survey of Health, Ageing and Retirement in Europe (SHARE).

## Data Availability

The datasets generated and/or analysed during the current study are not publicly available due restrictions imposed by part of the owners (COURAGE in Europe, the Health 2000 and 2011 Surveys- Finland), but may be available from the corresponding author upon reasonable request and once consent form ATHLOS project intellectual property and dissemination board is obtained.

## Abbreviations

ATHLOS: Ageing Trajectories of Health – Longitudinal Opportunities and Synergies
AUC: Area Under the Receiver Operating Curve
BMI: Body Mass Index
CHARLS: China Health and Retirement Longitudinal Study
COURAGE in Europe: Collaborative Research on Ageing in Europe
HRS: Health and Retirement Study
IQR: Interquartile ranges
MHAS: Mexican Health and Aging Study
OR: Odds ratio
SHARE: Survey of Health, Ageing and Retirement in Europe
SD: Standard deviation
VIF: Variance Inflation Factor
95% CI: 95% Confidence Intervals
