# Supplementary material for: Predictors of pain in general ageing populations: results from a multi-country analysis based on ATHLOS harmonized database
Source: J Headache Pain. 2020 May 6;21(1):45. doi: 10.1186/s10194-020-01116-3 (PMC7201730; doi:10.1186/s10194-020-01116-3)
Supplement: Supplementary file 1 — Additional file 1: Table S1. Description of exploited studies and waves. Table S2. Univariable logistic regression results. Table S3. Multivariable logistic regression results. Table S4. Descriptive statistics, China Health and Retirement Longitudinal Study (CHARLS). Table S5. Descriptive statistics Collaborative Research on Ageing in Europe Study (COURAGE in Europe). Table S6. Descriptive statistics Health and Retirement Study (HRS). Table S7. Descriptive statistics Health 2000/2011 study (Health 2000/2011). Table S8. Descriptive statistics Mexican Health and Aging Study (MHAS). Table S9. Descriptive statistics Survey of Health, Ageing and Retirement in Europe (SHARE). [file 10194_2020_1116_MOESM1_ESM.docx]

Supplementary materials.

Table S1. Description of exploited studies and waves.

| Study | Baseline wave | Follow-up wave | No pain  (N=8,197) | Yes pain  (N=5,348) | Total  (N=13,545) | Time between waves  (mean±sd [median: min - max]) |
| --- | --- | --- | --- | --- | --- | --- |
| CHARLS | W1 | W2 | 10.7% | 10.3% | 10.5% | 2.0±0.1 [2: 2-2] |
| COURAGE in Europe | W1 | W2 | 13.4% | 10.1% | 12.1% | 3.6±0.5 [4: 3-4] |
| HRS | W9 | W11 | 39.8% | 14.1% | 29.8% | 4.0 ± 0.2 [4: 4-4] |
| Health 2000/2011 | W1 | W2 | 15.1% | 11.6% | 13.7% | 10.9±0.4 [11: 11-11] |
| MHAS | W2 | W3 | 17.9% | 12.0% | 15.6% | 9.0±0.1 [9: 9-9] |
| SHARE | W4 | W5 | 3.1% | 41.9% | 18.2% | 2.0±0.0 [2: 2-2] |

Notes. CHARLS (China Health and Retirement Longitudinal Study); COURAGE in Europe (Collaborative Research on Ageing in Europe); HRS (Health and Retirement Study); Health 2000/2011 (Health 2000/2011 study); MHAS (Mexican Health and Aging Study); SHARE (Survey of Health, Ageing and Retirement in Europe)

Table S2: Univariable logistic regression results

| VARIABLES | Baseline OR  (95% CI) | Follow-up OR  (95% CI) | Variable definition |
| --- | --- | --- | --- |
| Female gender (ref: male) | 1.49 ***  (1.39-1.59) | 1.50***  (1.40-1.61) | Biological sex |
| Marital status (ref: married) |  |  |  |
| Single | 1.14  (0.95-1.38) | 1.40***  (1.15-1.70) | Current marital status |
| Divorced/separated | 1.19**  (1.01-1.40) | 1.18*  (1.00-1.40) |  |
| Widowed | 1.26***  (1.13-1.41) | 1.33***  (1.20-1.48) |  |
| Primary or less education level (ref: secondary or above) | 1.30***  (1.21-1.40) | 1.76***  (1.59-1.95) | Highest level of formal education achieved |
| Retired (ref: employed or student) | 1.17***  (1.08-1.26) | 1.01  (0.95-1.09) | Participant is retired |
| Wealth Quintiles (ref: Lower) |  |  |  |
| Quintile 2 | 0.84***  (0.75-0.94) | 1.15*  (0.98-1.34) | Total household wealth in quintiles |
| Quintile 3 | 0.81***  (0.72-0.92) | 0.89  (0.77-1.02) |  |
| Quintile 4 | 0.77***  (0.69-0.87) | 0.67***  (0.58-0.78) |  |
| Quintile 5 (Higher) | 0.67***  (0.59-0.75) | 0.62***  (0.53-0.72) |  |
| Smoking status (ref: never smoker) |  |  |  |
| Former smoker | 0.81***  (0.74-0.89) | 0.82***  (0.74-0.90) | Composite variable. Any type of tobacco smoking |
| Current smoker | 0.93*  (0.85-1.01) | 1.22***  (1.10-1.36) |  |
| Alcohol drinker (ref: non-drinker) | 0.84***  (0.78-0.90) | 0.86***  (0.80-0.92) | Current alcohol drinker |
| Engagement in vigorous exercise (ref: no up to moderate exercise) | 1.24***  (1.14-1.36) | 1.21***  (1.10-1.33) | Engage in vigorous exercise during the last 2 weeks |
| Low energy/Fatigue (ref: high level of energy) | 1.20***  (1.12-1.29) | 2.14***  (1.97-2.32) | Self-reported high level of energy |
| Sleep problems (ref: no problems) | 1.58***  (1.48-1.70) | 2.15***  (2.01-2.31) | Sleeping problems |
| Walking difficulties (ref: no difficulties)) | 1.63***  (1.45-1.82) | 2.54***  (2.32-2.78) | Mobility - walking. Difficulty for walking by yourself and without using any special equipment |
| Self-rated health (ref: good) |  |  |  |
| Averege/Fair/Moderate | 1.56***  (1.43-1.70) | 2.03***  (1.88-2.19) | Respondent's self-rated/self-reported health |
| Poor | 2.3***  (2.02-2.671) | 4.27***  (3.82-4.77) |  |
| Recent falls (ref: no recent falls) | 2.90***  (2.68-3.14) | 3.21***  (2.97-3.46) | Recent falls in the last 6-24 months |
| Well-being (ref: high) |  |  |  |
| Middle | 1.33***  (1.23-1.44) | 1.50***  (1.38-1.63) | Evaluative well-being |
| Low | 3.39***  (2.98-3.86) | 4.00***  (3.53-4.53) |  |
| Diabetes (ref: absence of disease) | 1.60***  (1.43-1.78) | 1.11*  (1.00-1.23) | History of diabetes mellitus |
| Respiratory diseases (ref: absence of disease) | 1.28***  (1.14-1.43) | 1.29***  (1.14-1.45) | History of chronic respiratory diseases such asthma, CPD, COPD, bronchitis, emphysema |
| Hypertension (ref: absence of disease) | 1.18***  (1.10-1.27) | 1.13***  (1.05-1.22) | History of hypertension |
| Joint disorders (ref: absence of disease) | 1.54***  (1.43-1.65) | 1.82***  (1.68-1.97) | History of arthritis, rheumatism or osteoarthritis |
| Angina (ref: absence of disease) | 1.45***  (1.18-1.78) | 1.59***  (1.28-1.98) | History of angina |
| Stroke (ref: absence of disease) | 1.82***  (1.36-2.43) | 1.43**  (1.03-1.98) | History of stroke |
| Cancer (ref: absence of disease) | 1.22*  (0.97-1.54) | 1.65***  (1.26-2.16) | Any malignancy (C00-D48) / information about past or current neoplasm (benign, in situ, malignant) |
| Multimorbidity (ref: one disease only) | 1.44***  (1.34-1.56) | 1.62***  (1.51-1.75) | Multimorbidity status |
| Obesity (ref: absence of disease) | 1.18***  (1.06-1.32) | 1.27***  (1.14-1.41) | Presence of obesity (BMI value ≥30Kg/m^2^). |
| Depression (ref: absence of disease) | 2.09***  (1.91-2.30) | 3.27***  (3.00-3.57) | Current depressive status |
| Bereavement (ref: no bereavement) | 1.51***  (1.40-1.63) | 2.37***  (2.20-2.55) | Experience of a loss of any close person |

Note: **p*<0.10, ** *p*<0.05, *** *p*<0.01

Table S3: Multivariable logistic regression results

| Models  Variables | Model A | Model B | Model C | Model D | Model E | Model F | Model G | Model H | Model I |
| --- | --- | --- | --- | --- | --- | --- | --- | --- | --- |
|  | OR  (95% CI) | OR  (95% CI) | OR  (95% CI) | OR  (95% CI) | OR  (95% CI) | OR  (95% CI) | OR  (95% CI) | OR  (95% CI) | OR  (95% CI) |
| Constant | 0.12***  (0.10-0.15) | 0.23***  (0.19-0.26) | 0.23***  (0.20-0.26) | 0.23***  (0.20-0.26) | 0.28***  (0.19-0.41) | 0.52***  (0.43-0.64) | 0.50***  (0.42-0.60) | 0.38***  (0.32-0.45) | 0.39***  (0.33-0.46) |
| Baseline Variables |  |  |  |  |  |  |  |  |  |
| Female gender | 1.38***  (1.19-1.61) | 1.39***  (1.24-1.56) | 1.39***  (1.24-1.57) | 1.41***  (1.27-1.58) | 1.29**  (1.03-1.63) | 1.36***  (1.18-1.56) | 1.35***  (1.18-1.55) | 1.32***  (1.16-1.49) | 1.34***  (1.18-1.51) |
| Engage in vigorous  exercise | 1.27***  (1.09-1.47) | 1.50***  (1.32-1.71) | 1.48***  (1.30-1.68) | 1.59***  (1.41-1.79) | 1.28*  (1.00-1.63) | 3.10***  (2.64-3.64) | 3.01***  (2.57-3.53) | 2.49***  (2.16-2.87) | 2.51***  (2.18-2.89) |
| Bereavement | 1.18**  (1.02-1.37) | 1.31***  (1.17-1.46) | 1.30***  (1.16-1.46) | 1.46***  (1.31-1.63) | 1.08  (0.86-1.35) | 1.90***  (1.64-2.20) | 1.88***  (1.63-2.18) | 1.84***  (1.61-2.09) | 1.88***  (1.65-2.15) |
| Obesity | 1.18**  (1.00-1.40) | 1.23***  (1.09-1.38) | 1.25***  (1.11-1.40) | 1.24***  (1.10-1.39) | 1.10  (0.82-1.47) | 1.17**  (1.00-1.36) | 1.18**  (1.01-1.37) | 1.34***  (1.16-1.54) | 1.36***  (1.18-1.57) |
| Self-rated health,  average to moderate (vs. good) | 1.93***  (1.56-2.40) | 1.65***  (1.44-1.89) | 1.67***  (1.46-1.91) | 1.71***  (1.50-1.94) | 1.09  (0.78-1.52) | 1.07  (0.89-1.28) |  |  |  |
| Self-rated health,  poor (vs. good) |  | 2.24***  (1.78-2.81) | 2.30***  (1.83-2.88) | 2.31***  (1.85-2.88) | 0.74  (0.19-2.89) | 0.95  (0.61-1.48) |  |  |  |
| Sleep problems | 1.64***  (1.41-.91) | 1.35***  (1.21-1.52) | 1.36***  (1.21-1.52) | 1.38***  (1.24-1.55) | 1.06  (0.82-1.37) | 1.25***  (1.07-1.46) | 1.24***  (1.06-1.44) | 1.12  (0.98-1.28) |  |
| Multimorbidity | 1.30**  (1.06-1.60) | 1.10  (0.98-1.24) |  |  |  |  |  |  |  |
| Smoking,  former smoker (vs. never) | 1.30***  (1.10-1.53) | 0.87**  (0.77-1.00) | 0.88*  (0.78-1.01) |  |  |  |  |  |  |
| Smoking,  current smoker (vs. never) | 1.67***  (1.38-2.02) | 1.15*  (1.00-1.33) | 1.15*  (0.99-1.33) |  |  |  |  |  |  |
| Follow-up Variables |  |  |  |  |  |  |  |  |  |
| Low energy/Fatigue |  |  |  |  | 2.19***  (1.69-2.85) | 1.97***  (1.67-2.32) | 1.94***  (1.65-2.27) | 1.93***  (1.68-2.21) | 1.93***  (1.68-2.22) |
| Self-rated health,  average to moderate (vs. good) |  |  |  |  | 3.57***  (2.53-5.03) | 2.32***  (1.94-2.78) | 2.28***  (1.93-2.69) | 1.56***  (1.35-1.81) | 1.57***  (1.36-1.82) |
| Self-rated health,  poor (vs. good) |  |  |  |  | 7.16***  (2.50-20.48) | 3.80***  (2.57-5.60) | 3.55***  (2.52-5.01) | 2.22***  (1.72-2.85) | 2.20***  (1.71-2.83) |
| Difficulties with walking |  |  |  |  | 3.34***  (2.07-5.40) | 1.52***  (1.26-1.83) | 1.50***  (1.25-1.81) | 1.64***  (1.40-1.93) | 1.69***  (1.43-1.99) |
| Sleep problems |  |  |  |  | 1.55***  (1.21-1.99) | 1.88***  (1.61-2.18) | 1.87***  (1.61-2.17) | 1.76***  (1.54-2.02) | 1.80***  (1.59-2.05) |
| Respiratory disease |  |  |  |  | 1.46**  (1.09-1.96) | 1.26**  (1.03-1.56) | 1.12  (0.91-1.38) |  |  |
| Joint disorders |  |  |  |  | 2.01**  (1.10-3.70) | 0.96  (0.83-1.11) |  |  |  |
| Stroke |  |  |  |  | 0.32**  (0.11-0.90) | 0.90  (0.54-1.52) |  |  |  |
| Observations | 4,578 | 16,097 | 16,098 | 16,979 | 2,664 | 11,852 | 11,980 | 13,430 | 13,545 |
| Hosmer-Lemeshow’s p-value |  | 0.0854 | 0.7947 | 0.0377 |  | 0.0103 | 0.0281 | 0.0114 | 0.0013 |
| Predicted p-value^§^ |  | <0.0001 | <0.0001 | <0.0001 |  | <0.0001 | <0.0001 | <0.0001 | <0.0001 |
| Predicted squared p-value^§^ |  | 0.115 | 0.096 | 0.319 |  | 0.019 | 0.128 | 0.141 | 0.208 |
| Akaike criterion (AIC) |  | 18956.02 | 18959.95 | 20506.97 |  | 12599.60 | 12813.68 | 15070.29 | 15216.07 |
| Bayesian criterion (BIC) |  | 19040.57 | 19036.81 | 20568.89 |  | 12725.06 | 12909.76 | 15160.35 | 15298.72 |
| AUC |  | 0.64  (0.63-0.65) | 0.64  (0.63-0.65) | 0.65  (0.64-0.66) |  | 0.79  (0.78-0.80) | 0.79  (0.78-0.80) | 0.77  (0.76-0.78) | 0.77  (0.76-0.78) |

Notes: *** p<0.01, ** p<0.05, * p<0.1, § for specification error check. All models were weighted and adjusted for time-lag variable.

Model A: Selected variable by stepwise forward logistic regressions with baseline variables only;

Model B: Multivariable logistic regression with selected stepwise predictors at Model A (corresponding to manuscript’s Model 1);

Model C: Refined Model B, with statistically significant only predictors (p<0.05), deleting multimorbidity variable;

Model D: Refined Model C, with statistically significant only predictors (p<0.05), deleting smoking variable (corresponding to manuscript’s Model 2);

Model E: Selected variable by stepwise forward logistic regressions with follow-up variables, with Model D significant variables;

Model F: Multivariable logistic regression with selected stepwise predictors at Model E (corresponding to manuscript’s Model 3);

Model G: Refined Model F, with statistically significant only predictors (p<0.05), deleting Joint disorders, Stroke and baseline Self-rated health variables;

Model H: Refined Model G, with statistically significant only predictors (p<0.05), deleting respiratory diseases;

Model I: Refined Model H, with statistically significant only predictors (p<0.05), deleting baseline sleep problems – Final model

Table S4: Descriptive statistics, China Health and Retirement Longitudinal Study (CHARLS)

|  | Baseline-W1  (N=1,454) | Missing data at baseline, n(%) | Follow-up-W2  (N=1,454) | Missing data at follow-up, n(%) |
| --- | --- | --- | --- | --- |
| Age, mean±sd  [median: interquartile range] | 60.8±10.3  [60: 53-68] |  | 62.8±10.3  [62: 55-70] |  |
| Presence of pain | - |  | 62.0% |  |
| Sex,  Male  Female | 41.0%  59.0% |  | 41.0%  59.0% |  |
| Marital status,  Married/cohabiting  Single  Divorced/Separated  Widow | 84.7%  0.6%  1.2%  13.5% |  | 83.1%  0.5%  0.9%  15.5% |  |
| Education,  Primary or less  Secondary or above | 73.5%  26.5% |  | 100%  0% | 1453 (99.93) |
| Retired | 12.5% | 27 (1.86) | 15.2% | 9 (0.62) |
| Household wealth quintile  1 - Lower  2  3  4  5 - Higher | 23.3%  20.3%  19.3%  17.0%  20.1% | 118 (8.12) | 36.4%  14.6%  20.7%  15.8%  12.5% | 578 (39.75) |
| Smoking status,  Never  Former smoker  Current | 65.6%  8.5%  25.9% |  | 93.5%  4.3%  2.2% | 498 (34.25) |
| Current alcohol consumption | 27.8% |  | 27.9% |  |
| Engaged in vigorous exercise | 30.4% |  | 29.6% | 203 (13.96) |
| Low level of energy/Fatigue | 49.5% | 17 (1.17) | 44.1% |  |
| Sleep problems | 48.0% | 9 (0.62) | 49.3% |  |
| Difficulty walking by yourself | 23.3% | 525 (36.11) | 25.1% |  |
| Self-reported health,  Poor  Average/Fair/Moderate  Good | 21.1%  53.1%  25.8% | 725 (49.86) | 30.9%  51.7%  17.4% |  |
| Recent falls | 12.2% | 1 (0.07) | 17.2% |  |
| Evaluative wellbeing,  Low  Middle  High | 12.4%  63.4%  24.2% | 119 (8.18) | 14.1%  62.7%  23.2% | 17 (1.17) |
| Diabetes | 6.4% | 8 (0.55) | 100% | 1322 (90.92) |
| Respiratory diseases | 12.1% | 8 (0.55) | 100% | 1361 (93.60) |
| Hypertension | 28.4% | 10 (0.69) | 100% | 924 (63.55) |
| Joint disorders | 28.8% |  | 100% | 864 (59.42) |
| Angina | - | 1454 (100) | - | 1454 (100) |
| Stroke | 2.1% | 3 (0.21) | 100% | 1429 (98.28) |
| Cancer | 0.7% | 5 (0.34) | 100% | 1445 (99.38) |
| Multimorbidity | 24.3% |  | 43.7% | 446 (30.67) |
| Obesity | 5.3% |  | 6.5% | 285 (19.6) |
| Depression | 34.2% | 15 (1.03) | 35.4% | 12 (0.83) |
| Bereavement | 28.9% |  | 22.0% |  |

Table S5: Descriptive statistics Collaborative Research on Ageing in Europe Study (COURAGE in Europe)

|  | Baseline-W1  (N=1,602) | Missing data at baseline, n(%) | Follow-up-W2  (N=1,602) | Missing data at follow-up, n(%) |
| --- | --- | --- | --- | --- |
| Age, mean±sd  [median: interquartile range] | 42.0±15.9  [39: 30-53] |  | 45.5±15.9  [43: 33-56] |  |
| Presence of pain | - |  | 67.4% |  |
| Sex,  Male  Female | 54.6%  45.4% |  | 54.6%  45.4% |  |
| Marital status,  Married/cohabiting  Single  Divorced/Separated  Widow | 57.5%  32.7%  5.5%  4.3% |  | 58.9%  28.7%  7.3%  5.1% |  |
| Education,  Primary or less  Secondary or above | 36.3%  63.7% | 1 (0.06) | 28.5%  71.5% |  |
| Retired | 13.0% | 131 (8.18) | 7.8% | 377 (23.53) |
| Household wealth quintile  1 - Lower  2  3  4  5 - Higher | 25.0%  13.3%  16.6%  21.5%  23.6% | 133 (8.30) | 27.6%  15.4%  15.2%  21.3%  20.5% | 637 (39.76) |
| Smoking status,  Never  Former smoker  Current | 45.9%  16.9%  37.2% |  | 30.8%  23.4%  45.8% | 374 (23.35) |
| Current alcohol consumption | 76.4% |  | 89.5% | 529 (33.02) |
| Engaged in vigorous exercise | 20.5% |  | 20.7% | 14 (0.87) |
| Low level of energy/Fatigue | 9.3% |  | 13.3% |  |
| Sleep problems | 15.2% |  | 20.7% |  |
| Difficulty walking by yourself | 3.9% |  | 11.4% |  |
| Self-reported health,  Poor  Average/Fair/Moderate  Good | 2.0%  15.3%  82.7% |  | 1.9%  15.3%  82.8% |  |
| Recent falls | - | 1602 (100) | - | 1602 (100) |
| Evaluative wellbeing,  Low  Middle  High | 2.0%  7.2%  90.8% |  | 5.7%  12.2%  82.1% |  |
| Diabetes | 4.9% |  | 7.1% | 1 (0.06) |
| Respiratory diseases | 5.4% |  | 7.2% | 1 (0.06) |
| Hypertension | 13.4% |  | 17.8% | 4 (0.25) |
| Joint disorders | 5.1% |  | 8.7% |  |
| Angina | 1.9% |  | 2.6% | 1 (0.06) |
| Stroke | 1.1% |  | 1.9% | 2 (0.12) |
| Cancer | - | 1602 (100) | 2.7% | 518 (32.33) |
| Multimorbidity | 6.7% |  | 10.5% |  |
| Obesity | 18.8% |  | 20.6% | 58 (3.62) |
| Depression | 4.5% |  | 2.6% | 1 (0.06) |
| Bereavement | 4.3% |  | 5.1% |  |

Table S6: Descriptive statistics Health and Retirement Study (HRS)

|  | Baseline  (N=3,998) | Missing data at baseline, n(%) | Follow-up  (N=3,998) | Missing data at follow-up, n(%) |
| --- | --- | --- | --- | --- |
| HRS | W9 |  | W11 |  |
| Age, mean±sd  [median: interquartile range] | 70.1±5.3  [70: 67-74] |  | 74.2±5.2  [74: 71-78] |  |
| Presence of pain | - |  | 81.5% |  |
| Sex,  Male  Female | 43.4%  56.6% |  | 43.4%  56.6% |  |
| Marital status,  Married/cohabiting  Single  Divorced/Separated  Widow | 71.3%  2.3%  9.0%  17.3% |  | 65.7%  2.5%  9.0%  22.8% | 1 (0.03) |
| Education,  Primary or less  Secondary or above | 15.8%  84.2% |  | 15.8%  84.2% |  |
| Retired | 62.9% | 398 (9.95) | 73.3% | 167 (4.18) |
| Household wealth quintile  1 - Lower  2  3  4  5 - Higher | 13.0%  18.0%  21.0%  23.4%  24.6% |  | 15.2%  18.8%  20.8%  21.6%  23.6% |  |
| Smoking status,  Never  Former smoker  Current | 42.4%  47.3%  10.3% |  | 42.4%  48.8%  8.8% | 1 (0.03) |
| Current alcohol consumption | 54.8% |  | 53.3% |  |
| Engaged in vigorous exercise | 3.1% |  | 2.7% | 12 (0.30) |
| Low level of energy/Fatigue | 11.1% | 36 (0.90) | 14.0% |  |
| Sleep problems | 21.6% | 33 (0.83) | 22.1% |  |
| Difficulty walking by yourself | 14.0% | 33 (0.83) | 23.7% |  |
| Self-reported health,  Poor  Average/Fair/Moderate  Good | 2.6%  12.1%  85.3% | 2 (0.05) | 3.8%  14.3%  81.9% |  |
| Recent falls | 27.3% | 434 (10.86) | 28.2% | 200 (5.00) |
| Evaluative wellbeing,  Low  Middle  High | - | 3998 (100) | - | 3998 (100) |
| Diabetes | 18.6% | 3 (0.08) | 24.2% | 3 (0.08) |
| Respiratory diseases | 7.9% | 2 (0.05) | 11.2% | 4 (0.10) |
| Hypertension | 57.1% | 5 (0.13) | 65.6% | 6 (0.15) |
| Joint disorders | 55.9% | 5 (0.13) | 63.4% | 10 (0.25) |
| Angina | - | 3998 (100) | - | 3998 (100) |
| Stroke | 1.0% | 6 (0.15) | 1.3% | 7 (0.18) |
| Cancer | 1.8% | 12 (0.30) | 2.3% | 15 (0.38) |
| Multimorbidity | 52.3% | 1 (0.03) | 63.8% | 1 (0.03) |
| Obesity | 28.7% |  | 27.9% | 27 (0.68) |
| Depression | 6.2% | 159 (3.98) | 7.6% |  |
| Bereavement | 17.3% |  | 22.8% | 1 (0.03) |

Table S7: Descriptive statistics Health 2000/2011 study (Health 2000/2011)

|  | Baseline  (N=1,852) | Missing data at baseline, n(%) | Follow-up  (N=1,852) | Missing data at follow-up, n(%) |
| --- | --- | --- | --- | --- |
| Health 2000/2011 | W1 |  | W2 |  |
| Age, mean±sd  [median: interquartile range] | 45.7±10.8  [45: 36-53] |  | 56.7±10.8  [56: 47-64] |  |
| Presence of pain | - |  | 67.0% |  |
| Sex,  Male  Female | 47.7%  52.3% |  | 47.7%  52.3% |  |
| Marital status,  Married/cohabiting  Single  Divorced/Separated  Widow | 78.1%  11.2%  8.2%  2.5% |  | 75.5%  8.6%  9.9%  6.0% |  |
| Education,  Primary or less  Secondary or above | 32.0%  68.0% |  | 29.7%  70.3% |  |
| Retired | 11.3% |  | 32.8% |  |
| Household wealth quintile  1 - Lower  2  3  4  5 - Higher | 9.6%  17.7%  18.7%  28.2%  25.8% | 37 (2.00) | - | 1852 (100) |
| Smoking status,  Never  Former smoker  Current | 36.3%  30.6%  33.1% | 309 (16.68) | 27.8%  52.9%  19.3% |  |
| Current alcohol consumption | 92.7% | 3 (0.16) | 88.6% | 6 (0.32) |
| Engaged in vigorous exercise | 69.4% |  | 100% | 1413 (76.3) |
| Low level of energy/Fatigue | 30.8% | 45 (2.43) | 30.4% |  |
| Sleep problems | 39.0% | 41 (2.21) | 49.2% |  |
| Difficulty walking by yourself | 1.5% |  | 7.3% |  |
| Self-reported health,  Poor  Average/Fair/Moderate  Good | 0.8%  12.6%  86.6% |  | 2.6%  11.0%  86.4% |  |
| Recent falls | - | 1852 (100) | 100% | 1649 (89.04) |
| Evaluative wellbeing,  Low  Middle  High | 0.9%  3.0%  96.1% | 60 (3.24) | 0.7%  2.9%  96.4% | 24 (1.30) |
| Diabetes | 2.2% | 2 (0.11) | 7.1% |  |
| Respiratory diseases | 14.1% | 1 (0.05) | 19.3% | 91 (4.91) |
| Hypertension | 19.8% |  | 31.8% |  |
| Joint disorders | 0.4% | 1 (0.05) | 1.2% |  |
| Angina | 1.6% |  | 2.2% |  |
| Stroke | 0.6% |  | 1.5% | 5 (0.27) |
| Cancer | 2.8% |  | 3.9% | 43 (2.32) |
| Multimorbidity | 6.6% |  | 15.2% |  |
| Obesity | 15.5% |  | 21.3% | 1 (0.05) |
| Depression | 4.2% | 7 (0.38) | 4.5% | 47 (2.54) |
| Bereavement | 62.4% |  | 6.0% |  |

Table S8: Descriptive statistics Mexican Health and Aging Study (MHAS)

|  | Baseline  (N=2,148) | Missing data at baseline, n(%) | Follow-up  (N=2,148) | Missing data at follow-up, n(%) |
| --- | --- | --- | --- | --- |
| MHAS | W2 |  | W3 |  |
| Age, mean±sd  [median: interquartile range] | 62.9±7.3  [62: 57-68] |  | 72.2±7.3  [71: 66-77] |  |
| Presence of pain | - |  | 70.0% |  |
| Sex,  Male  Female | 52.3%  47.7% |  | 52.3%  47.7% |  |
| Marital status,  Married/cohabiting  Single  Divorced/Separated  Widow | 65.6%  3.9%  6.8%  23.7% |  | 54.5%  4.7%  7.5%  33.3% |  |
| Education,  Primary or less  Secondary or above | - | 2148 (100) | - | 2148 (100) |
| Retired | 12.3% |  | 23.0% | 3 (0.14) |
| Household wealth quintile  1 - Lower  2  3  4  5 - Higher | 18.4%  19.7%  19.9%  24.8%  17.2% | 111 (5.17) | 21.2%  23.7%  23.1%  17.6%  14.4% | 295 (13.73) |
| Smoking status,  Never  Former smoker  Current | 59.4%  22.9%  17.7% |  | 56.5%  30.2%  13.3% | 1 (0.05) |
| Current alcohol consumption | 28.7% |  | 24.6% | 1 (0.05) |
| Engaged in vigorous exercise | 34.4% |  | 32.8% | 1 (0.05) |
| Low level of energy/Fatigue | 58.8% | 2 (0.09) | 50.0% |  |
| Sleep problems | 30.4% |  | 34.3% |  |
| Difficulty walking by yourself | 10.3% | 12 (0.56) | 28.9% |  |
| Self-reported health,  Poor  Average/Fair/Moderate  Good | 7.4%  46.4%  46.2% |  | 11.5%  49.8%  38.7% |  |
| Recent falls | 24.7% |  | 41.3% | 2 (0.09) |
| Evaluative wellbeing,  Low  Middle  High | - | 2148 (100) | 4.5%  10.7%  84.8% | 7 (0.33) |
| Diabetes | 14.1% | 2 (0.09) | 25.8% | 3 (0.14) |
| Respiratory diseases | 6.8% | 3 (0.14) | 9.7% | 2 (0.09) |
| Hypertension | 42.1% | 3 (0.14) | 61.3% |  |
| Joint disorders | 17.5% | 1 (0.05) | 24.4% | 1 (0.05) |
| Angina | - | 2148 (100) | - | 2148 (100) |
| Stroke | 0.2% | 1 (0.05) | 2.0% | 16 (0.74) |
| Cancer | 0.03% | 4 (0.19) | 1.0% | 19 (0.88) |
| Multimorbidity | 20.0% |  | 38.1% |  |
| Obesity | 18.9% |  | 33.1% | 1855 (87.76) |
| Depression | 25.5% | 3 (0.14) | 31.4% |  |
| Bereavement | 40.2% |  | 99.9% | 3 (0.14) |

Table S9: Descriptive statistics Survey of Health, Ageing and Retirement in Europe (SHARE)

|  | Baseline  (N=2,491) | Missing data at baseline, n(%) | Follow-up  (N=2,491) | Missing data at follow-up, n(%) |
| --- | --- | --- | --- | --- |
| SHARE | W4 |  | W5 |  |
| Age, mean±sd  [median: interquartile range] | 63.7±9.8  [62: 55-71] |  | 65.6±9.8  [64: 57-73] |  |
| Presence of pain | - |  | 10.2% |  |
| Sex,  Male  Female | 44.9%  55.1% |  | 44.9%  55.1% |  |
| Marital status,  Married/cohabiting  Single  Divorced/Separated  Widow | 71.9%  7.5%  7.6%  13.0% | 16 (0.64) | 70.4%  7.7%  7.7%  14.2% | 18 (0.72) |
| Education,  Primary or less  Secondary or above | 33.9%  66.1% | 12 (0.48) | 33.9%  66.1% | 13 (0.52) |
| Retired | 43.9% | 5 (0.20) | 48.2% | 15 (0.60) |
| Household wealth quintile  1 - Lower  2  3  4  5 - Higher | 22.0%  19.1%  16.7%  21.1%  21.1% | 6 (0.24) | 22.3%  21.9%  17.9%  18.7%  19.2% | 8 (0.32) |
| Smoking status,  Never  Former smoker  Current | 51.2%  23.3%  25.5% | 562 (22.56) | 0.8%  0.1%  99.1% | 2042 (81.98) |
| Current alcohol consumption | 62.4% | 1 (0.04) | 62.5% | 1 (0.04) |
| Engaged in vigorous exercise | 50.4% |  | 47.9% | 1 (0.04) |
| Low level of energy/Fatigue | 32.3% | 32 (1.28) | 43.0% |  |
| Sleep problems | 31.4% | 32 (1.28) | 38.0% |  |
| Difficulty walking by yourself | 7.0% | 1 (0.04) | 11.5% |  |
| Self-reported health,  Poor  Average/Fair/Moderate  Good | 7.5%  22.9%  69.6% |  | 11.4%  32.2%  56.4% |  |
| Recent falls | 97.2% |  | 91.4% |  |
| Evaluative wellbeing,  Low  Middle  High | 34.1%  31.4%  34.5% | 42 (1.69) | 42.6%  33.7%  23.7% | 32 (1.28) |
| Diabetes | 33.0% | 1602 (64.31) | 18.5% | 2 (0.08) |
| Respiratory diseases | 6.6% |  | 9.8% | 2 (0.08) |
| Hypertension | 35.6% |  | 47.3% | 2 (0.08) |
| Joint disorders | 19.5% |  | 43.4% | 2 (0.08) |
| Angina | - | 2491 (100) | - | 2491 (100) |
| Stroke | 3.3% |  | 2.9% | 2 (0.08) |
| Cancer | 5.5% |  | 5.8% | 2 (0.08) |
| Multimorbidity | 23.8% |  | 39.8% | 2 (0.08) |
| Obesity | 22.7% |  | 21.1% | 44 (1.77) |
| Depression | 25.3% | 59 (2.37) | 34.3% | 34 (1.36) |
| Bereavement | 61.5% |  | 93.5% | 5 (0.20) |
